# The association between human papillomavirus infection and female lung cancer: A population-based cohort study

**DOI:** 10.1097/MD.0000000000003856

**Published:** 2016-06-10

**Authors:** Frank Cheau-Feng Lin, Jing-Yang Huang, Stella Ching-Shao Tsai, Oswald Ndi Nfor, Ming-Chih Chou, Ming-Fang Wu, Chun-Te Lee, Cheng-Feng Jan, Yung-Po Liaw

**Affiliations:** a School of Medicine, Chung Shan Medical University; b Department of Thoracic Surgery, Chung Shan Medical University Hospital; c Department of Public Health and Institute of Public Health, Chung Shan Medical University; d Department of Medical Research, Tungs’ Taichung Metro Harbor Hospital; e Department of Food and Nutrition, Providence University; f Divisions of Medical Oncology and Pulmonary Medicine, Chung Shan Medical University Hospital, Taichung; g Department of Psychiatry, Chung Shan Medical University Hospital; h Chung Yuan Christian University, Taoyuan City; i Department of Family and Community Medicine, Chung Shan Medical University Hospital, Taichung, Taiwan.

**Keywords:** cohort, human papillomavirus, lung cancer, population-based

## Abstract

Lung cancer is the leading cause of cancer deaths among Taiwanese women. Human papillomavirus (HPV) has been detected in lung cancer tissues. The aim of this study was to investigate the association between HPV infection and lung cancer among the Taiwanese women. The analytical data were collected from the longitudinal health insurance databases (LHID 2005 and 2010) of the National Health Insurance Research Database (NHIRD). The study participants were 30 years and older and included 24,162 individuals who were identified with HPV infection from 2001 to 2004 and 1,026,986 uninfected individuals. Lung cancer incidence among infected and uninfected individuals was compared using the univariate and multivariate regression models. Among the total participants, 24,162 individuals were diagnosed with HPV. After adjusting for age, gender, low income, residential area, and comorbidity, the risk of lung cancer was higher in women (hazard ratio [HR] 1.263, 95% CI 1.015–1.571), while all cancer risks were high in both men and women with corresponding hazard ratios (HR) of 1.161 (95% CI 1.083–1.245) and HR 1.240 (95% CI 1.154–1.331), respectively. This study showed a significant increase in lung cancer risk among Taiwanese women who were exposed to HPV infection.

## 1 Introduction

Cancer has been reported as one of the leading causes of deaths among the Taiwanese since 1982 with approximately 20 to 30 deaths/100,000/ year.^[1]^ The etiologies of cancer have been widely studied with genetic and environmental factors proven to play a major role. Viral infection is one of the most significant risk factors for cancer. Studies conducted in Taiwan have reported associations between hepatitis B virus infection and hepatocellular carcinoma. On the other hand, nasopharyngeal cancer has been linked with Epstein–Barr virus.^[2]^ Human papillomavirus has been shown to have a causal relationship with cervical cancer.^[3]^ However, immunization against the virus has been introduced within the public health sector.^[1,4–6]^

Lung cancer is the leading cause of cancer death among Asian women particularly the Taiwanese.^[1,7]^ The most investigated and frequent cause of lung cancer is tobacco smoking.^[8]^ However, the incidence of female lung cancer has increased even without a concurrent increase in smoking among Asian populations.^[7]^ From 1981 to 2011, the female-to-male ratio for lung cancer increased rapidly from 1:3.3 to 1:2. Furthermore, a 3.3-fold increase in lung cancer death has been observed among Taiwanese women.^[1,9]^ The number of deaths due to cancer was 646 per 8.6 million in 1981 and 2782 per 11.5 million in 2010. Lung cancer incidence among female smokers in Taiwan was approximately 4% (2.3%–5.2%) in 1974.^[10,11]^ The cancer types were predominantly adenocarcinoma, often with epidermal growth factor receptor (EGFR) mutations, and which responded to treatment with tyrosine-kinase inhibitors.^[8,12]^ Lung cancer among female nonsmokers may be regarded as a different disease entity and therefore, etiology other than smoking needs to be investigated. HPV has been reported in lung cancer tissues in Western and Eastern countries especially Asia. Types 16 and 18 have been the most common pathogenic species.^[13–16]^ HPV has also been found in the breast tissue, head, neck, esophageal, and anogenital regions.^[4,17–20]^

A single-payer national health insurance was initiated in Taiwan since 1995 and has a coverage rate of about 99%. A nationwide population-based cohort study for cancer has been proven to be a reliable method to study the etiology of cancer.^[21–23]^ The aim of this study was to investigate the relationship between HPV and lung cancer among Taiwanese women.

## 2 Methods

**Figure 1:**
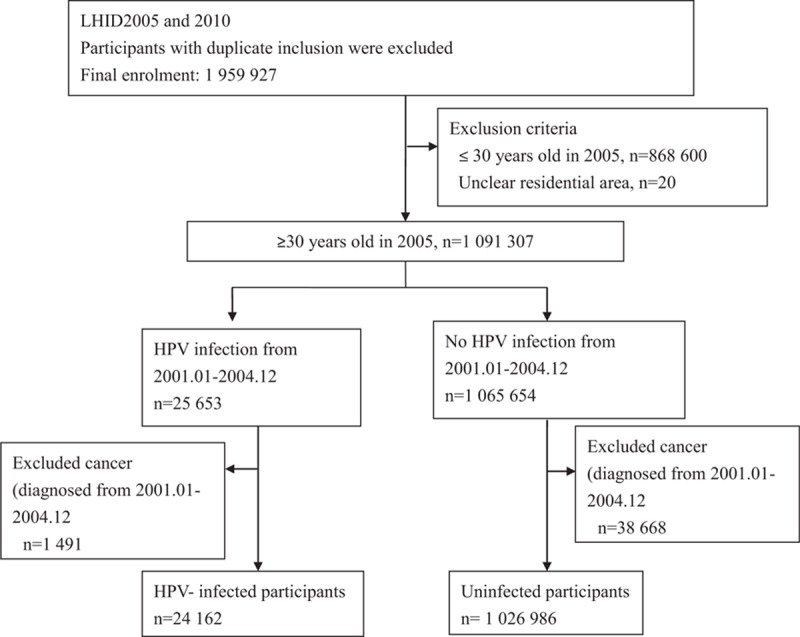
Flowchart of the study population.

The longitudinal health insurance databases (LHID 2005 and 2010) were used to collect data. Initially enrolled in this study were 25,653 individuals who were identified with HPV infection and 1,065,654 control individuals aged 30 years and older. Excluded were patients diagnosed with cancer from January 2001 to December 2004 and people aged 30 years and younger. Finally, 24,162 HPV-infected and 1,026,986 uninfected individuals were enrolled in the study (Fig. 1). HPV was identified using the International Classification of Diseases Clinical Modification (ICD-9-CM) codes 079.4, 078.1, 078.10–078.12, 078.19, 759.05, 795.09, 795.15, 795.19, 796.75 and 796.79.

The codes 140 to 208 were used for all cancer while 162 was for lung cancer. The crude incidence rate (per 1000 person-months) was calculated while the incidence rate ratio (IRR) with 95% confidence interval (CI) was estimated by Poisson regression. A 2-tailed *t* test was used to compare the mean difference between continuous variables while the *χ*^2^ test was used for nominal variables. For the time to event analysis of the longitudinal follow-up, the event was defined as the date of cancer onset. Follow-up was censored when a patient quit the insurance or in a case of death. The Kaplan–Meier survival curves and multiple Cox regression model were used to calculate the relative risk of cancer. SAS software (version 9.3, SAS Institute Inc, Cary, NC) was used for analysis. A *P* <0.05 was considered statistically significant. The study was approved by the Institutional Review Board of Chung Shan Medical University Hospital, Taichung, Taiwan (#CS13168).

## 3 Results

**Figure 2:**
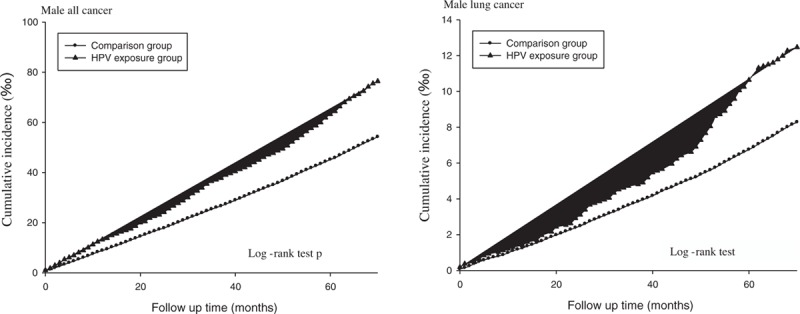
Kaplan–Meier curve of the cumulative incidence of lung cancer among infected and uninfected male participants.

**Figure 3:**
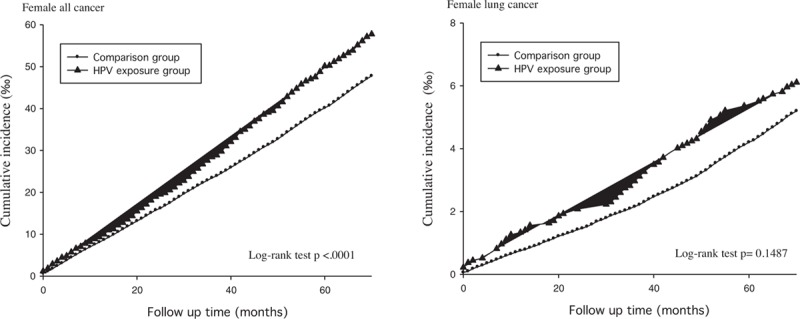
Kaplan–Meier curve of the cumulative incidence of cancer among infected and uninfected female participants.

The prevalence of HPV infection was 2.30%. Table 1 shows the basic demographic characteristics of the study population. There was no significant age difference between the HPV-infected individuals (48.61 ± 14.06 years) and their uninfected counterparts (48.73 ± 13.65 years). The exposed or infected individuals had a significantly higher proportion of females (55.85%), less low income (0.45%), and higher proportions of specific comorbidities (including chronic obstructive pulmonary disease (COPD), ischemic heart disease, hypertension, diabetes mellitus, liver disease, and renal disease). Table 2 shows the incidence rates (per 1000 person-months) for all cancer and lung cancer and the IRR of the HPV-infected and uninfected individuals stratified by gender. The mean follow-up times of the infected and uninfected male participants were 67.72 and 67.88 months, respectively. The IRR for all types of cancer among the infected males was 1.423 (95% CI: 1.328–1.526), while that for lung cancer was 1.518 (95% CI: 1.278–1.802). Similarly, the IRR for all cancer among the infected females was 1.208 (95% CI: 1.125–1.297), whereas that for lung cancer was 1.181(95% CI: 0.949–1.469). Figures 2 and 3 show the cumulative incidence rates for all types of cancer and lung cancer stratified by gender, while Tables 3 and 4 show the multiple Cox regression analysis of HPV infection and cancer. Adjustments were made for age, low-income household, residential area, and comorbidity. Results showed that HPV infection was significantly related to all cancer in men. Among males with HPV infection, the HR for all types of cancer and lung cancer were 1.161 (95% CI: 1.083–1.245) and 1.169 (95% CI: 0.984–1.39), respectively. The independent risk factor for male lung cancer was COPD, followed by diabetes mellitus, and liver disease. In females, HPV infection was significantly related to all types of cancer as well as lung cancer. Among females with HPV infection, the HR for all types of cancer and lung cancer were 1.240 (95% CI: 1.154–1.331) and 1.263 (95% CI: 1.015–1.571), respectively. COPD was the most significant independent risk factor for female lung cancer followed by HPV infection and liver diseases. After adjusting for the factors mentioned above, HPV infection was associated with lung cancer risk in females.

**Table 1:**
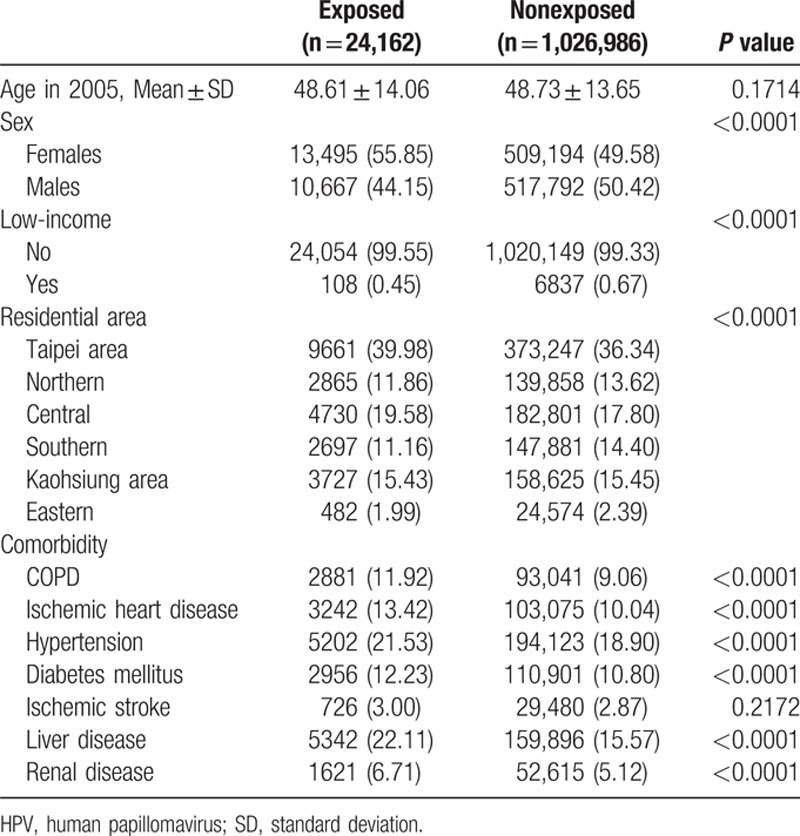
Demographic characteristics of HPV-exposed and nonexposed individuals in 2005.

**Table 2:**
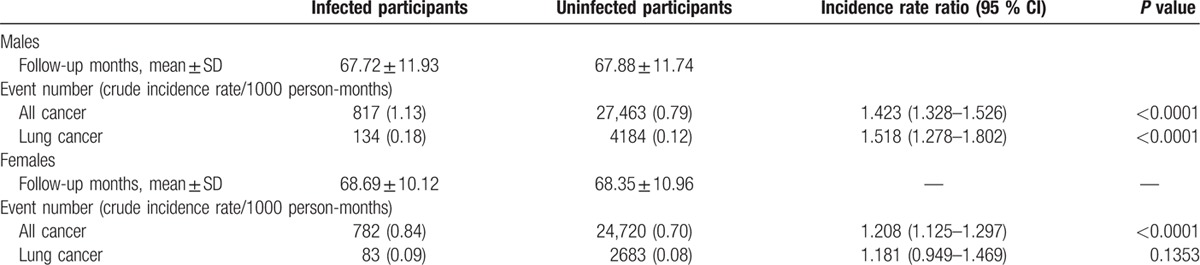
Cancer incidence among the infected and uninfected participants from 2005 to 2010∗.

**Table 3:**
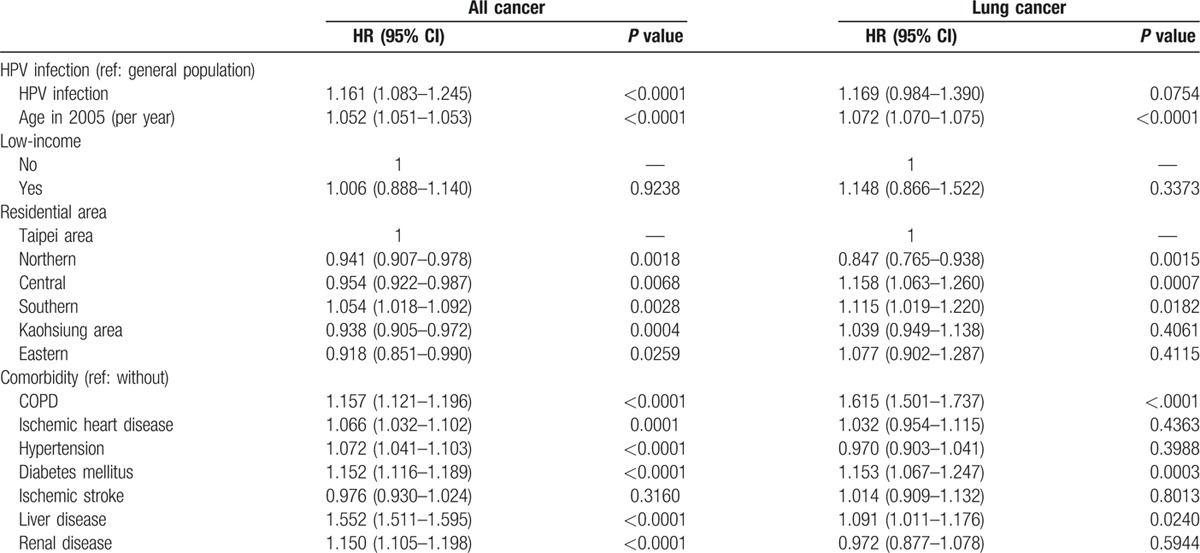
Cox regression analysis of HPV and lung cancer in males.

**Table 4:**
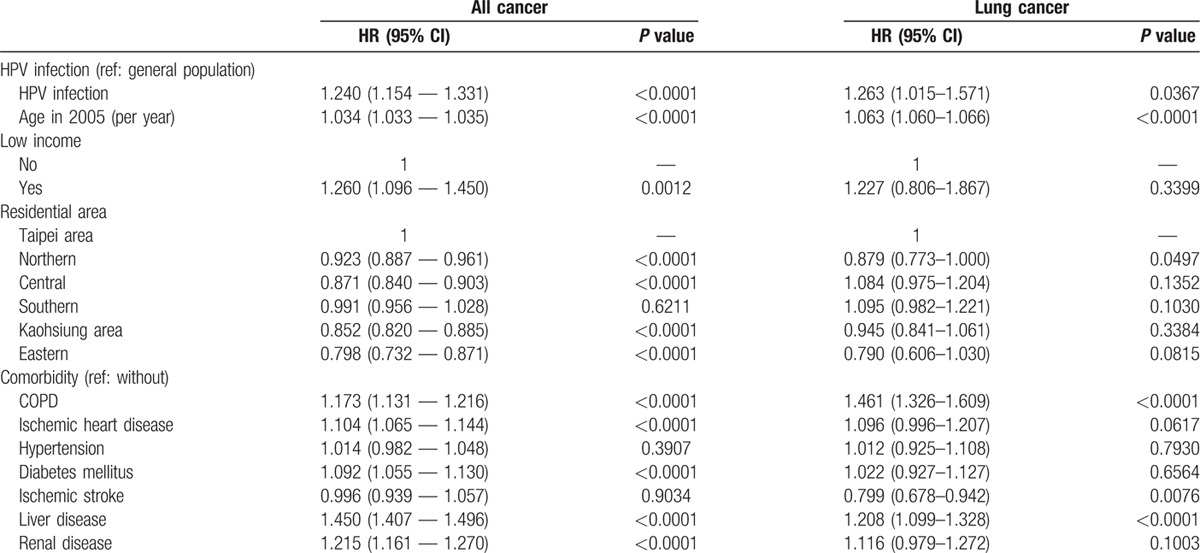
Cox regression analysis of HPV and lung cancer in females.

## 4 Discussion

In this study, a significant increase in lung cancer risk was found in females with HPV infection. Cigarette smoking, the modifiable risk factor for lung cancer was not adjusted due to a dearth of information in the database. However, adjustments were made for COPD and smoking-induced diseases. From 2005 to 2010, all cancer incidence rates were higher in the exposed males and females. Similar results have been reported.^[4,17–20]^

COPD was the major risk factor for lung cancer in both males and females. Jian et al^[24]^ have reported similar findings among lung cancer patients in Taiwan. Adjustments were also made for ischemic heart disease and hypertension associated with smoking^[25]^ as well as diabetes mellitus which has also been associated with all cancer.^[26,27]^ Surprisingly, liver disease was found to be an independent risk factor for lung cancer in this study. Hepatitis C infection has been implicated in the causation of lung cancer.^[28,29]^ Liver disease especially chronic viral hepatitis has been associated with hepatocellular carcinoma. Hepatocellular carcinoma is capable of metastasizing to the lung through the inferior vena cava. More studies are required to clarify such mechanisms.

The leading cause of lung cancer is cigarette smoking.^[8,30,31]^ Other suspected risk factors include passive smoking and cooking oil fumes, especially among Chinese women.^[31–33]^ In China, indoor air pollution has been associated with lung cancer.^[34]^ Air pollution has also been recognized recently as a carcinogen, especially for adenocarcinoma in females though the concept has long been suggested.^[35,36]^ The incidence of cancer has been attributed to the presence of heavy metals such as arsenic in soil and water.^[37,38]^ These risk factors would not be identified by analysis of the ICD-9-CM designations used in this study.

There is an extensive literature regarding HPV association with laryngeal and cervical cancer but HPV is also associated with other cancers, including lung cancer. Women with anogenital malignancy and HPV have been reported with a higher relative risk of lung cancer.^[39,40]^ HPV has been detected in lung cancer tissue since 1979.^[4,41,42]^ It was first detected in squamous cell carcinoma. The morphological characteristic was similar to that of condyloma and cervical cancer. In a meta-analysis, 22.4% of lung cancer tissues particularly adenocarcinoma and squamous cell carcinoma had HPV predominantly types 16 and 18.^[43]^ Other pathological types of HPV have also been investigated. In Taiwan, HPV 16/18 DNA has been detected in 54.6% of lung cancer tissues whereas the surrounding normal lung tissues were spared. The odds ratio for HPV 16/18 infection of lung cancer tissue in female nonsmokers was about 4 and 11 times more than that of the male smokers and was about 2-fold higher in adenocarcinoma than squamous cell carcinoma.^[44]^

Transmission of HPV to the lungs is another issue of interest. HPV DNA and mRNA have been detected in the peripheral blood of patients with cervical cancer.^[45,46]^ The implication was that the HPV went through the bloodstream to the heart, and then to the lung. Lungs are reticuloendothelial system rich organs which may capture the virus. It is also possible that the HPV DNA/mRNA detected in the blood may have originated from metastasized cancer cells. However, this could not be clarified in this study because the data could not be separated by the site of HPV infection. In another study, HPV 16/18 DNA was detected in 47.7% of the blood from patients with lung cancer. The odds ratios for lung cancer in patients with HPV 16/18 ranged from 6 to 10.^[47]^ The virus is able to spread from superficial sites of the body via blood to the periphery of the lungs where they are trapped. The peripheral sites of the lung are prone to adenocarcinoma. This might explain why HPV-infected female nonsmokers were more likely to have adenocarcinoma.^[48]^ HPV has also induced adenocarcinoma in cervix.^[49]^ Other transmission routes include the foregut through the airway to the lung. Transmission of HPV from the oral cavity to the lungs has also been reported.^[50]^ The HPV E6 oncoprotein reduces the tumor suppressor p53 through the E6-associated protein-mediated ubiquitin pathway and results in tumorigenesis in cervical cancer.^[51,52]^ This mechanism was also observed in lung cancer.^[53]^ The HPV E7 oncoprotein promotes degradation of tumor suppressors Rb family via the ubiquitin-proteasomal-mediated pathway and interferes with the relationship of Rb family protein and E2F transcription family, hence inducing tumorigenesis. The damages of retinoblastoma-Cyclin D1-p16 cell cycle pathway are important in the carcinogenesis of lung cancer.^[54]^

This study did not particularly focus on adenocarcinoma. The study limitations include the following: first, the NHIRD does not contain information about life-style, smoking status, and smog, hence adjustments could not be made for such variables. Second, the database does not provide detailed ICD-9- CM codes for oral, pharynx, or laryngeal papilloma. This makes it difficult to compare the incidence of oral and laryngeal papilloma with lung cancer based on the ICD-9-CM. Third, although HPV types 16, 18, and 31 are risk factors for lung cancer, the pathologic types could not be determined using the ICD-9 CM code. Lastly, there is a possibility of a coding error in the administrative datasets. The strength of this study lies in the use of a large database. In addition, there was a complete follow-up, potential bias due to small sample sizes and limited demographics were reduced. In conclusion, this study suggests that HPV infection is an important risk factor for lung cancer among women in Taiwan. COPD was the most significant independent risk factor lung cancer. Vaccination against HPV can theoretically prevent lung cancer. However, future studies ought to focus on the effectiveness of HPV vaccine in preventing lung cancer.
